# Influences of Pre-Existing Fissure Angles and Bridge Angles on Concrete Tensile Failure Characteristics: Insights from Meshless Numerical Simulations

**DOI:** 10.3390/ma17174305

**Published:** 2024-08-30

**Authors:** Cong Hu, Taicheng Li, Zhaoqing Fu, Haiying Mao, Siyao Wang, Zilin Liang, Shuyang Yu

**Affiliations:** 1 China Renewable Energy Engineering Institute, Beijing 100120, China; 0538hucong@163.com (C.H.); litc@creei.cn (T.L.); fuzq@creei.cn (Z.F.); 2 School of Civil and Architectural Engineering, Guangxi University of Science and Technology, Liuzhou 545006, China; 3 Key Laboratory of Disaster Prevention & Mitigation and Prestress Technology of Guangxi Colleges and Universities, Liuzhou 545006, China; 4 School of Transportation and Civil Engineering, Nantong University, Nantong 226019, China; wangsiyao@ntu.edu.cn (S.W.); 19517111903@163.com (Z.L.)

**Keywords:** concrete meso-structures, crack propagation, SPH, numerical simulation

## Abstract

The existence of cracks is a key factor affecting the strength of concrete. However, traditional numerical methods still have some limitations in the simulation of crack growth in fissured concrete structures. Based on this background, the numerical treatment method of particle failure in smoothed particle hydrodynamics (SPH) is proposed, and the generation method for concrete meso-structures under the smoothed particle hydrodynamics (SPH) framework is developed. The concrete meso-models under different pre-existing micro-fissure inclinations and bridge angles (the inner tip line of the double pre-existing micro-fissure is defined as a bridge, and the angle between the bridge and the horizontal direction is defined as the bridge angle) were established, and numerical simulations of the crack propagation processes of concrete structures under tensile stress were carried out. The main findings were as follows: The concrete meso-structures and the pre-existing micro-fissures all have great impacts on the final failure modes of concrete. The stress–strain curve of the concrete model presents four typical stages. Finally, the crack initiation and propagation mechanisms of fissured concrete are discussed, and the application of smoothed particle hydrodynamics (SPH) in crack simulations of fissured concrete is prospected.

## 1. Introduction

Concrete material has the advantages of strong plasticity, various strength grades and economic durability, so it has been widely used in water conservancy projects. However, due to the low tensile strength of concrete itself, various types of initial cracks will occur in hydraulic concrete structures under the influences of temperature change, foundation settlement, steel corrosion or overload. For example, the Zeuzier arch dam [1] in Switzerland has many parallel bank slope cracks on the downstream surface of its body. Furthermore, in the initial stage of the construction of the Xiaowan arch dam [2], there were some parallel cracks in some sections. In addition, the Sayanshushensk arch dam [3] in the former Soviet Union produced many different types of cracks during its construction and operation, including cracks with different angles. When cracks exist in a concrete structure, the integrity and rigidity of the structure is affected. During the operation of a structure, various types of initial cracks in the structure will further expand under the influence of crack interaction under complex loads (especially when tensile forces are provided by water pressure). Meanwhile, they will form a series of pathologies (such as concrete carbonation and leakage corrosion damage, etc.), which will lead to the shortening of the service life of the structure, and even cause structure failure. It can also be found from the investigations of the working state of dams conducted by the International Dam Construction Commission in 1988 that the problem of the cross-propagation of cracks is the key factor in the failure of concrete dams. Therefore, the research on the interactive propagation behavior of cracks in concrete is the theoretical basis for the optimal design and safety performance evaluation of hydraulic structure engineering.

The numerical simulation method has the advantages of good repeatability, low cost, and comprehensive data. It can explain test phenomena from the internal mechanisms and further guide the design of tests. Therefore, as supplements for and extensions of test results, numerical simulations have been widely used in the study of crack propagation behavior and fracture performance in concrete structures [4,5,6,7]. Since the 1960s, domestic and foreign scholars have conducted a large amount of research on the numerical simulation of crack propagation processes [8], and they have proposed a variety of numerical models and simulation methods. In terms of the geometric modeling of cracks, the main approaches are the discrete fracture model, which characterizes discontinuity by separating the fracture surface, and the diffusion fracture model, which replaces single fractures based on continuum mechanics with distributed fractures. In terms of traditional numerical methods, the main approaches include the finite element method (FEM) and the boundary element method (BEM) [9,10,11]. Among the discrete crack models, the cohesive crack models (CCM) established by Barenblatt [12], Dugdale [13], and Hillerborg et al. [14] in ductile and quasi-brittle materials are becoming increasingly popular. This model describes the stiffness degradation by establishing the traction separation relationship between the element surfaces. It can not only simulate macroscopic cracks with strong discontinuity, but also describe the energy dissipation in the processes of viscous crack propagations. At the same time, it can also be pre-inserted into the initial grid as a cohesive element. The model can be realized using the finite element method or the boundary element method. In general, there are two main methods that can be used to model discrete viscous crack propagation: The first method is based on a complex mesh redivision program. For problems in which the crack propagation path is not known in advance, appropriate crack propagation criteria are needed to determine when and in which direction the crack will expand, which usually involves the calculation of the stress intensity factor (SIF) and the crack tip stress, the accuracy of which can only be guaranteed by fine crack tip meshes or the use of singular elements. Therefore, with the propagation of cracks, the grid needs to change accordingly [15,16]. At the same time, it is difficult to deal with complex crack growth situations, such as multi-crack and three-dimensional problems, based on grid-based repartition. Another method is to pre-insert or pre-embed cohesive elements in the initial finite element mesh [17,18,19]. This method does not require crack propagation criteria or mesh redivision, so complex crack propagation can be simulated. In view of this, increasing numbers of scholars at home and abroad have combined cohesive crack models (CCMs) with the finite element method to simulate the softening characteristics of the concrete fracture process zone (FPZ) and the expansion of cracks. Santiago et al. [20] simulated the cracking behavior of concrete around steel bars caused by corrosion by embedding two-dimensional cohesive elements, and the results showed that the finite element model proposed in their paper could predict the time taken for surface cracking quite accurately. By inserting cohesive elements into initial finite elements such as solid elements in a batch, Su et al. [21] realized the simulation of complex crack propagation in concrete wedge-split-tensile and torsional fracture models under static and dynamic loads with the finite element software ABAQUS, and compared the simulated fracture path, *P*-CMOD curve, and other fracture parameters with the test results. Good consistency was obtained, and the effectiveness of the method was verified. At the same time, in addition to the traditional finite element and boundary element simulation methods, the extended finite element method (XFEM) [22,23,24] has become increasingly popular in crack propagation modeling in recent years. Based on the idea of unit decomposition [25], the extended finite element method (XFEM) introduces the step function and the crack tip strengthening function into the displacement interpolation function, which not only obtains macroscopic crack propagation, but also overcomes the problem that the crack path is required to be consistent with the element boundary in the discrete crack model, and avoids the shortcoming that the grid needs to be redivided after crack propagation. Therefore, this method can simulate crack propagation well. However, the extended finite element method (XFEM) has some limitations in dealing with multi-crack propagation and interaction.

The smoothed particle hydrodynamic method (SPH) is a meshless method. The computational domain is characterized by a series of regularly distributed particles, and the mechanical parameters of the particles are calculated by the governing equations. Compared with the finite element method, it is not necessary to carry out mesh regeneration with this method, so it can better adapt to the heterogeneous and discontinuous characteristics of concrete damage evolution simulations. In addition, the parameters of SPH are strictly derived from partial differential equations, and their parameters have clear physical meaning, avoiding complex parameter calibrations. So far, the SPH method has been applied to a certain extent in the simulation of rock fracture processes. For example, Zhou’s groups proposed the so-called general particle dynamics (GPD) method [26,27,28] based on the SPH method, making it possible to simulate the whole process of rock fracture initiation, expansion, and failure. However, the application of the SPH method to simulate the mesoscopic damage evolution of fissured concrete has not been reported.

Aiming at the shortcomings of previous studies, the numerical simulation method based on SPH was developed to simulate the whole process of the meso-tensile failure of fissured concrete. Firstly, according to the parameter transfer mode in the traditional SPH governing equation, a numerical processing method that can reflect the damage of SPH particles is proposed. Next, we develop a meso-structure generation algorithm for random aggregates, random pores, and their transition layers, which can generate a meso-structure model of concrete with controllable meso-components in SPH. Subsequently, a concrete mesoscopic model with different pre-existing micro-fissure angles and double fissure bridging angles is established to simulate crack propagation and evolution under uniaxial tension. Finally, the crack initiation mechanisms and the double fissure interaction mechanisms are discussed in detail. The research results can provide a certain reference for applications of the SPH method in the simulation of concrete meso-cracking.

## 2. SPH Foundation Property Equations and Fracture Treatment Method

### 2.1. SPH Fundamental Property Equations

Kernel function approximation method and particle approximation method are the two cores of SPH calculation. The aim of the kernel function approximation method is to approximate the value and derivative of the continuous field quantity by discrete sampling points (particles), while the aim of the particle approximation method is to apply the discretized particles in SPH to further approximate the nuclear approximation equation, by applying the values corresponding to adjacent particles in the local region to superposition the sum instead of the integral of the field function and its derivative. The expression of kernel function approximation can be written as follows:

(1) $$f(x)=\int_{\Omega }f(x^{\prime })\delta (x-x^{\prime })dx^{\prime }$$

In the kernel function approximation expression (1), ***x*** is the particle coordinate vector; ***x***′ is the target particle coordinate vector; *f* represents the field function, which is used to represent variables such as density and velocity; *Ω* is the calculation domain of SPH; and *δ* represents the Dirac function. In SPH, the smoothed kernel function *W* is generally used to replace the Dirac function, so the expression of the field function is obtained [29], as follows:

(2) $$f(x)\approx \int_{\Omega }f(x^{\prime })W(x-x^{\prime },\;h)dx^{\prime }$$

The particle approximation in SPH can be expressed as follows [29]:

(3) $$f(x_{i})=\sum_{j=1}^{N}\frac{m_{j}}{\rho_{j}}f(x_{j})\cdot W_{ij}$$

where *m_j_* is the mass of SPH particles and *ρ_j_* is the density of SPH particles.

In SPH, the parameters of each particle are calculated and the results are visualized through the governing equation, which includes the continuity equation and the momentum equation, whose expression can be written as follows [29]:

(4) $$\frac{d\rho_{i}}{dt}=\sum_{j=1}^{N}m_{j}v_{ij}^{\beta }\partial W_{ij,\beta }$$

(5) $$\frac{dv_{i}^{\alpha }}{dt}=\sum_{j=1}^{N}m_{j}(\frac{\sigma_{ij}^{\alpha \beta }}{\rho_{i}^{2}}+\frac{\sigma_{ij}^{\alpha \beta }}{\rho_{j}^{2}}+T_{ij})\partial W_{ij,\beta }$$

In the governing Equations (4) and (5), *ρ* and *m* are the density and mass of the particle, respectively. *v* and *σ* are the velocity and stress of the particle, respectively. The subscripts of the parameters *i* and *j* represent the serial number of the particle. *t* stands for the time parameter. *N* is the total number of SPH particles. *α* and *β* are Einstein notations. *W* is the smoothing kernel function.

### 2.2. SPH Fracture Treatments

To deal with the fracture processes in SPH, it is necessary to determine its fracture criterion first. In this section, the improved Mohr–Coulomb criterion commonly used in previous numerical simulations is adopted, and its expression is as follows [30]:

(6) $$\sigma_{1}=\sigma_{t}$$

(7) $$\tau_{f}=c+\sigma_{f}\tan\varphi$$

In the fracture criteria (6) and (7), *σ*_1_ is the maximum principal stress on the SPH particle; *σ_t_* is the tensile strength of the SPH particle; *φ* is the internal friction angle of particles in SPH; and *σ_f_* and *τ_f_* are tensile stress and shear stress on the failure surface of the material, respectively.

**Figure 1:**
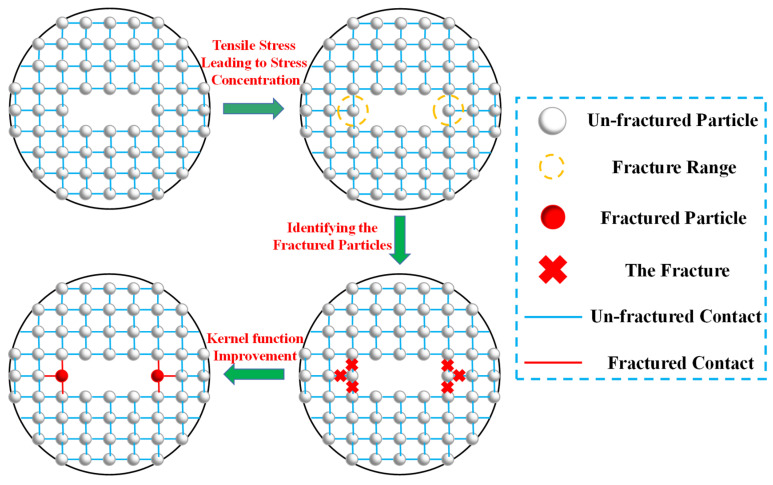
Numerical treatments of particle fracture in SPH.

In order to characterize the particle failure in SPH, a fracture marker *χ* is introduced in this section, according to the key parameter transfer rules in the SPH governing equation, and its initial value is set to 1, indicating that the SPH particle has not broken. When the stress on the SPH particle reaches the fracture criteria in Equation (6) or (7), the fracture marker *χ* = 0 is defined. This indicates that the SPH particle is broken, and the connection between the broken particle and the surrounding particles is cut off. The particle fracture treatment in SPH is shown in Figure 1. At the same time, the improved smooth kernel function is defined as *I*, and the relationship between the improved smooth kernel function *I* and the traditional smooth kernel function *W* in SPH can be expressed as follows [28]:

(8) $$I(x-x^{\prime },\;h)=\chi \cdot W(x-x^{\prime },\;h)$$

where *I* is the improved smooth kernel function; *χ* is the fracture marker; and *W* is the original kernel function.

By substituting the improved smoothed kernel function *I* into the governing Equations (4) and (5), the SPH governing equation considering particle fracture can be obtained as follows [28]:

(9) $$\frac{d\rho_{i}}{dt}=\sum_{j=1}^{N}m_{j}v_{ij}^{\beta }\partial I_{ij,\beta }$$

(10) $$\frac{dv_{i}^{\alpha }}{dt}=\sum_{j=1}^{N}m_{j}(\frac{\sigma_{ij}^{\alpha \beta }}{\rho_{i}^{2}}+\frac{\sigma_{ij}^{\alpha \beta }}{\rho_{j}^{2}}+T_{ij})\partial I_{ij,\beta }$$

where *ρ* and *m* are the density and mass of the particle. *v* and *σ* are the velocity and stress of the particle, respectively. The subscripts of the parameters *i* and *j* represent the serial number of the particle. *t* stands for the time parameter. *N* is the total number of SPH particles. *α* and *β* are Einstein notations. *W* is the smoothing kernel function.

## 3. SPH Generation Methods of Aggregates, Pores, and Transition Layers

### 3.1. Determination Method for Mesoscopic Parameters of Aggregates, Pores and Transition Layers

The meso-structures of concrete mainly consist of mortar, aggregate, porosity, and the transition layer. The differences in the compositions of concrete meso-structures are the root causes of concrete’s heterogeneity, which has a great influence on its crack expansions and damage evolutions. In order to correctly characterize the meso-structures of concrete, it is first necessary to determine the generation algorithm of the aggregate, pores, and mortar in concrete. The key of the generation algorithm is to ensure that pores do not coincide with the aggregate and the transition zone. The specific generation steps are as follows:

(1) Determine the model parameters. According to the requirements of the numerical simulation, the size of the model is determined. For the two-dimensional condition, determine the length *l* and width *m* of the model. At the same time, according to the composition statistics of each meso-structure of the concrete model, it is determined that the percentage of aggregate is *P_a_* and the percentage of porosity is *P_v_*.
(2) The center coordinates (*xa_i_*, *ya_i_*) of the random circular aggregate and the radius *ra_i_* of the circular aggregate are generated by setting random number seeds. Before generation, it is necessary to compare the generated circular aggregate *i* with the previously generated circular core aggregate *i* − 1 to make sure that the *i*th circular aggregate does not overlap with the previous *i* − 1 circular aggregates. The principle of determination is that the center distance between the *i*th circular aggregate and the *i* − 1 circular aggregates must be greater than the sum of the radii of the *i* circular aggregate and the *i* − 1 circular aggregate. For each circular aggregate that is determined to be generated, the particles around the periphery are the transition layer particles. For each *i*th circular aggregate generated, determine whether the ratio of the area of 1~*i* aggregates to the model area *l* × *m* has reached the predetermined percentage *P_a_*. If so, step (3) is performed; if not, step (2) is repeated.
(3) The center coordinates (*xv_i_*, *yv_i_*) of random pores are generated by setting random number seeds, and the radius of circular pore is *rv_i_*. Before the formation, it is necessary to compare the generated circular pore *i* with the previously generated *i* − 1 circular center pores to make sure that the *i*_th_ circular pore does not overlap with the former *i* − 1 circular pores and the circular aggregates generated in step (2). The determination principle is that the distance between the center of the circles of the *i*th circular pore and the *i* − 1th circular pore must be greater than the sum of the radius of the *i*th circular pore and the *i* − 1th circular pore. For each circular center pore *i* generated, determine whether the ratio of the area of 1~*i* circular pores to the model area l × m has reached the predetermined percentage *P_v_*. If so, perform step (4); if not, repeat step (3).
(4) Enter the generated concrete mesoscopic model parameters into the model input files.

### 3.2. SPH Meso-Structure Generation Processes

The SPH meso-structure generation adopts the “search circle method”, that is, for each meso-structure, determine whether SPH particles are covered in the corresponding circle area, so as to accurately identify the internal characteristics of concrete, such as aggregates, pores, transition layers, etc. The specific steps are as follows:

(1) Generate SPH matrix particles first, and their regular distributions are in the *l* × *m* range of the model. The parameters of the concrete pore structure generated in Section 3.1 are introduced. If SPH matrix particles are within the circle of the concrete pore, then no SPH particles are generated there, indicating that this place is a pore.
(2) Generate aggregate particles. Import the aggregate parameters in Section 3.1. If SPH matrix particles are within the circle of concrete pores, then SPH particles there are set as aggregate particles.
(3) Generate transition layer particles. Import the parameters of the transition layer in Section 3.1. If the SPH matrix particle is within the range of the transition layer, the SPH particle there is set as the transition layer particles.
(4) Different material mechanical parameters are assigned to different concrete meso-structures, and finally the concrete meso-SPH model is generated.

## 4. Numerical Simulation Results

### 4.1. Numerical Model and Parameters

**Figure 2:**
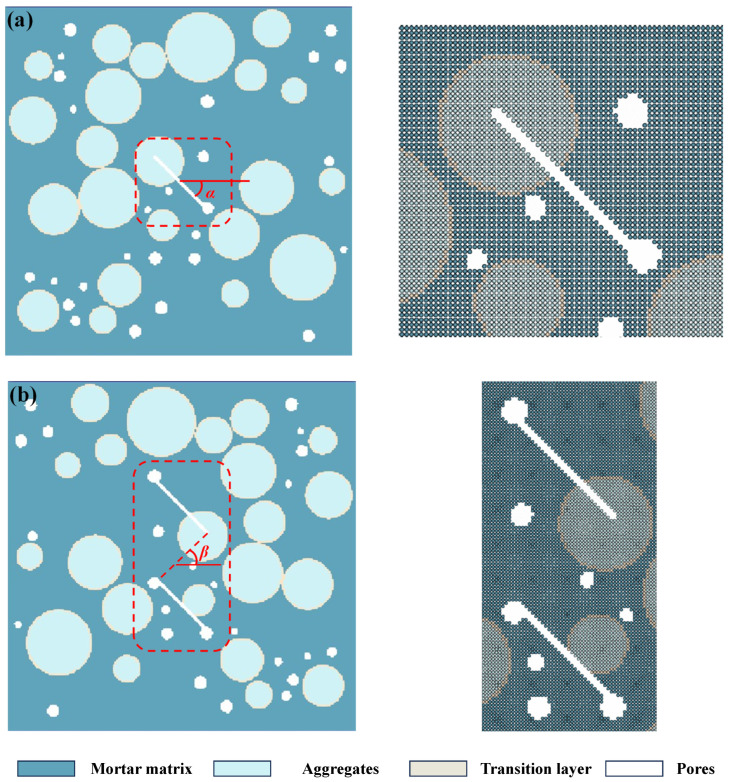
Numerical model and particle division of fissured concrete. (**a**) meso-model of concrete with single fissure; (**b**) meso-model of concrete with double fissures.

In order to explore the evolution mechanisms of concrete damage under tensile load and the influences of different fissure inclinations, fissure numbers, and fissure bridge angles on concrete meso-failure morphologies, a meso-concrete model is established, as shown in Figure 2, where Figure 2a is a concrete model with a single fissure, and Figure 2b is a concrete model with double fissures. The model size is 50 mm × 50 mm, the concrete aggregate percentage *P_a_* = 25%, and the concrete porosity percentage *P_v_* = 5%. The particle size of the aggregate ranges from 3 mm to 10 mm, while the diameter of the pores ranges from 1 mm to 2 mm. The length of the pre-existing micro-fissure is 10 mm. The angle between the pre-existing micro-fissure and the horizontal direction is defined as *α*. The inner tip line of the double pre-existing micro-fissure is defined as a bridge, and the angle between the bridge and the horizontal direction is defined as *β*. The uniaxial tensile displacement boundary is applied to the upper and lower side of the model. The numerical schemes are set as follows: (1) The different prefabricated crack inclination angles are A1: *α* = 0°, A2: *α* = 30°, A3: *α* = 45°, A4: *α* = 60°, and A5: *α* = 75°; and (2) the different double fissure bridge angles are B1: *β* = 0°, B2: *β* = 30°, B3: *β* = 45°, B4: *β* = 60°, and B5: *β* = 75°. The upper and lower boundaries of the model are subjected to uniaxial tensile action, and the loading rate is 0.005 m/s. The whole model is divided into 200 × 200 = 40,000 particles. The mesoscopic parameters of the model are set as follows [31]: the elastic modulus of the mortar matrix *E* = 25 GPa, the Poisson ratio of the mortar matrix *μ* = 0.2, the cohesion of the mortar matrix *c* = 5.95 MPa, the internal friction angle of the mortar matrix *φ* = 40°, and the tensile strength of the mortar matrix *σ_t_* = 2 MPa. The elastic modulus of the aggregate *E* = 70 GPa, the Poisson ratio of the aggregate *μ* = 0.2, and it is assumed that the aggregate will not be damaged. The elastic modulus of the transition layer *E* = 25 GPa, the Poisson ratio of the transition layer *μ* = 0.2, the cohesion of the transition layer *c* = 5.95 MPa, the internal friction angle of the transition layer *φ* = 40°, and the tensile strength of the transition layer *σ_t_* = 1 MPa. The average time consumed for one model is about 70 h.

### 4.2. Numerical Simulation Results Analysis

#### 4.2.1. Progressive Crack Propagation Processes

**Figure 3:**
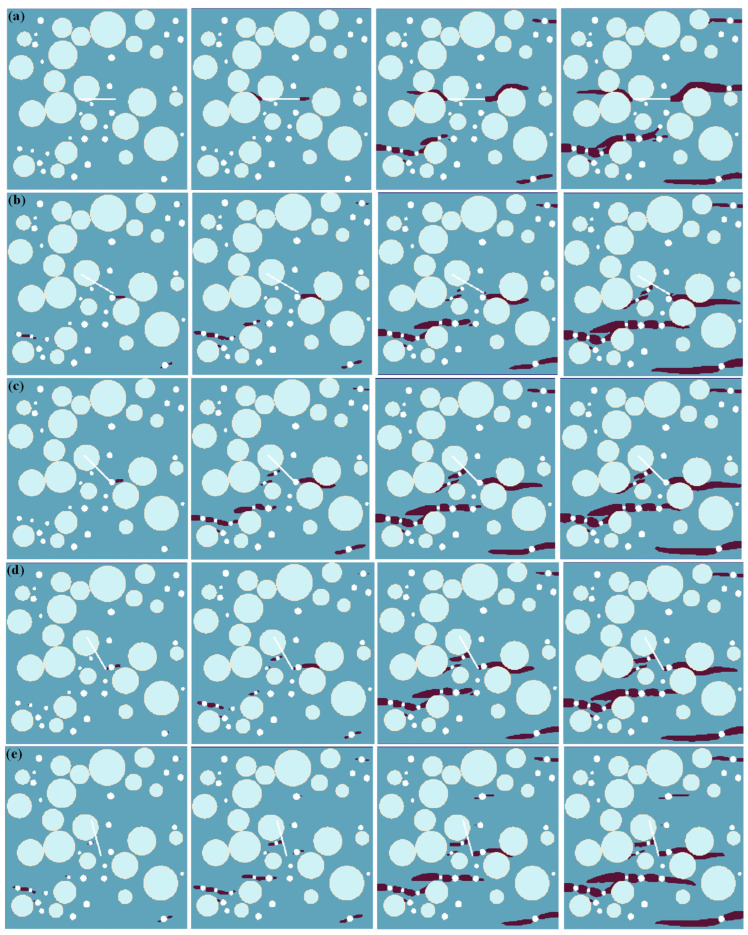
Numerical simulation results of tensile failure processes of meso-concrete under different pre-existing micro-fissure angles. (**a**) *α* = 0°; (**b**) *α* = 30°; (**c**) *α* = 45°; (**d**) *α* = 60°; (**e**) *α* = 75°.

Figure 3 shows the numerical simulation results of the tensile failure processes of the meso-concrete under different single-fissure inclination angles. It can be seen from the figure that the weak areas in the concrete meso-structure are the places where crack propagation easily occurs, such as pores, interfacial transition areas, and pre-existing micro-fissures. With the change in the position and inclination angle of the pre-existing micro-fissure, the stress distributions in the concrete structure are changed, thus leading to changes in the crack expansion morphologies. (1) When the inclination angle of the pre-existing micro-fissure is *α* = 0°, the crack propagation first occurs from the two tips of the pre-existing micro-fissure, and then “bypasses” the aggregate and extends approximately along the horizontal direction. After the crack generated at the pre-existing micro-fissure extends to a certain length, crack propagation occurs in the pores in the concrete structure. At the same time, the cracks generated by the pores overlap with each other to form large cracks. Finally, the large cracks formed by the pores and the horizontal cracks in the pre-existing micro-fissures combine to cause model failure. (2) When the inclination of the pre-existing micro-fissure is *α* = 30°, the upper left end of the pre-existing micro-fissure is in the concrete aggregate, which inhibits its crack expansion, and the lower right end of the pre-existing micro-fissure is close to a pore of the concrete. Unlike *α* = 0°, in this case, the crack is first generated from the pore instead of the pre-existing micro-fissure. When the crack in the pore extends to a certain length, a small segment of crack growth occurs at the lower right end of the pre-existing micro-fissure. However, the large crack generated by the pore eventually extends to the model boundary in the horizontal direction, resulting in the failure of the model. (3) When the inclination of the pre-existing micro-fissure is *α* = 45°, the upper left end of the pre-existing micro-fissure is also in the concrete aggregate, and its crack growth is inhibited, while the lower right end of the pre-existing micro-fissure is connected with the pores in the concrete structure, which is equivalent to forming a fissure-and-pore combination. The crack initiates at the lower right end of the fissure–pore combination. After the crack expands to a certain length, the cracks in the pore area expand and become a large crack. Finally, the crack growth in the fissure–pore combination and the crack growth in the pore area together lead to the failure of the model. (4) When the inclination angle of the pre-existing micro-fissure is *α* = 60°, the upper end of the pre-existing micro-fissure is located inside the aggregate, while the lower end is no longer bonded with the pores. Under the action of uniaxial tensile load, the pre-existing micro-fissure first generates crack growth and connects with the pores. Subsequently, crack growth occurs in other pores in the concrete structure. The crack growth in the pre-existing micro-fissure and the crack growth in the pore lead to the failure of the model. (5) When the inclination angle of the pre-existing micro-fissure is *α* = 75°, the crack does not initiate from the pre-existing micro-fissure due to the large inclination angle of the pre-existing micro-fissure, and the crack propagation occurs at the pore position first. In particular, the crack generated in the pore near the lower right end of the pre-existing micro-fissure is bonded with the pre-existing micro-fissure, and finally, the crack generated in the pore forms a large horizontal crack through the model and leads to failure.

**Figure 4:**
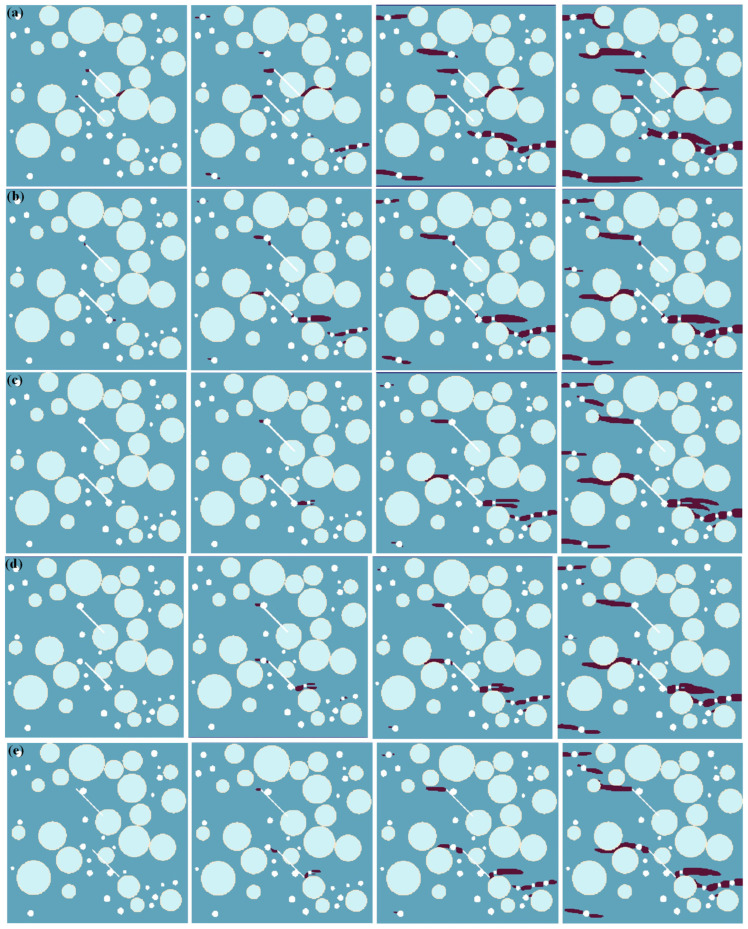
Numerical simulation results of tensile failure processes of meso-concrete under different bridge angles. (**a**) *β* = 0°; (**b**) *β* = 30°; (**c**) *β* = 45°; (**d**) *β* = 60°; (**e**) *β* = 75°.

Figure 4 shows the numerical simulation results of the tensile failure processes of meso-concrete under different double-fissure bridge angles. As can be seen from the figure, double fissures further change the stress distributions of the concrete structure under the uniaxial tensile force, so the failure mode is different from that in the case of single fissures. (1) When the bridge angle is *β* = 0°, the lower right end of the lower pre-existing micro-fissure is inside the aggregate. Therefore, the two ends of the upper pre-existing micro-fissure and the upper left end of the lower pre-existing micro-fissure first produce crack growth. When the crack in the three tips of the pre-existing micro-fissure extends to a certain length, the crack growth then occurs in the surrounding pores. Finally, the crack bonding in the pores and the crack propagation in the prefabricated double cracks together form horizontal crack penetration, which leads to the model’s failure. (2) When the bridge angles are *β* = 30° and 45°, the upper end of the pre-existing micro-fissure is close to a pore, and the lower end is inside the aggregate, so the crack propagation is inhibited. The upper and lower sections of the lower pre-existing micro-fissure are bonded with a pore, so the upper end of the upper pre-existing micro-fissure first produces crack growth and pore bonding, and the cracks in the pre-existing micro-fissure–pore combination formed by the two tips of the lower pre-existing micro-fissure and two pores are generated from the pore and expand along the horizontal direction. When the crack extends to a certain length, other pores in the concrete model begin to initiate. Finally, the pre-existing micro-fissure–pore combination and horizontal cracks in the pores together lead to the failure of the model. (3) For the bridge angle of *β* = 60°, the upper end of the upper pre-existing micro-fissure overlaps with a pore, and the lower end is inserted into the aggregate, the upper end of the lower pre-existing micro-fissure is close to a pore, and the lower end overlaps with a pore, but its tip is not inserted into the pore. Cracks are first generated from the pores connected to the upper end of the upper pre-existing micro-fissure and the two tips of the lower pre-existing micro-fissure, and then spread along the horizontal direction. Finally, the horizontal crack propagation generated by the pre-existing micro-fissures and pores leads to the failure of the model. (4) When the bridge angle is *β* = 75°, the lower end of the upper pre-existing micro-fissure overlaps with the aggregate, and both ends of the lower pre-existing micro-fissure are inside the concrete matrix. Cracks are first generated from the pre-existing micro-fissure tips, the crack in the upper tips of the pre-existing micro-fissure extends to the aggregate and then stops, the crack in the lower pre-existing micro-fissure tip extends to the pore, and the crack at the rear extends from the pore to the model boundary, finally leading to the failure of the model.

#### 4.2.2. Stress–Strain Curve Analysis

**Figure 5:**
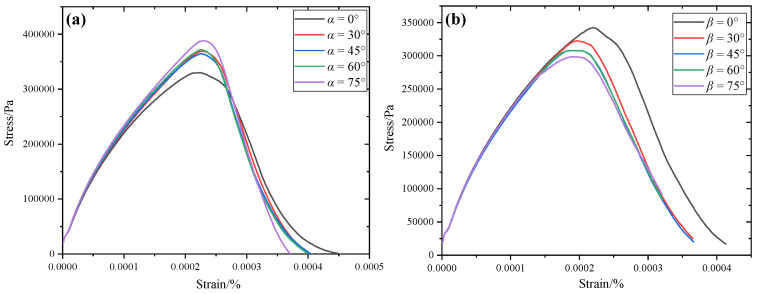
Stress–strain curve of meso-concrete during tensile processes. (**a**) single-fissure condition; (**b**) double-fissure condition.

Figure 5 shows the stress–strain curves with single and double fissures. As can be seen from the figure, the stress–strain curve for the concrete presents the same laws as in previous experimental studies, and there are four typical stages. (1) The linear elastic stage: in this stage, the stress and strain present linear variations, and no cracks occur inside the model. (2) The crack propagation stage: in this stage, cracks start to initiate, and the slope of the stress–strain curve becomes smaller, indicating that the elastic modulus gradually decreases. (3) The failure stage: the stress at this stage reaches its peak, indicating that the sample is about to fail. (4) The post-peak stage: in this stage, the strain increases, the stress gradually decreases, the crack propagation degree is large, and the sample damage is intensified.

For different pre-existing micro-fissure inclinations *α*, the peak strength of the concrete model gradually increases with the increase in *α*. This is because the stress concentration at both ends of the pre-existing micro-fissure decreases with the increase in the pre-existing micro-fissure inclination angle. At the same time, the upper end of the pre-existing micro-fissure falls into the aggregate with the increase in the pre-existing micro-fissure inclination angle, so the concrete strength gradually increases. For the different bridge angles, the strength of the concrete decreases gradually with the increase in the bridge angle *β*.

## 5. Discussions

### 5.1. Initiation Mechanisms of Fissured Concrete under Single-Fissure Condition

**Figure 6:**
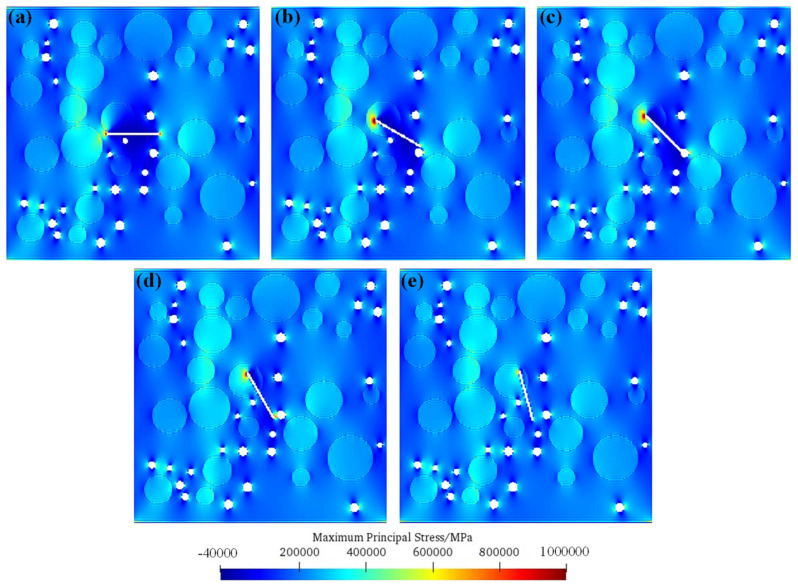
Maximum principal stress distributions before crack initiation in single fissure with different inclination angles. (**a**) *α* = 0°; (**b**) *α* = 30°; (**c**) *α* = 45°; (**d**) *α* = 60°; (**e**) *α* = 75°.

Figure 6 shows the maximum principal stress distributions before crack initiation for the single fissure with different inclination angles. As can be seen from the figure, when the inclination angle of the pre-existing micro-fissure is *α* = 0°, the tensile stress at both ends of the pre-existing micro-fissure is concentrated, indicating that the tensile crack occurs from both ends of the pre-existing micro-fissure. At the same time, it is worth noting that the tensile stress concentration at both ends of the pre-existing micro-fissure is the largest, except when the precast fissure tip is inside the aggregate, which indicates that the concrete model is more prone to cracking at this time, so its strength is lower. With the increase in the inclination angle of the pre-existing micro-fissure, the tensile stress concentration of the lower end of the pre-existing micro-fissure or the fissure–pore combination gradually decreases, indicating that the concrete model is more difficult to crack at this time, so its strength is higher.

### 5.2. Initiation Mechanisms of Fissured Concrete under Double-Fissure Condition

**Figure 7:**
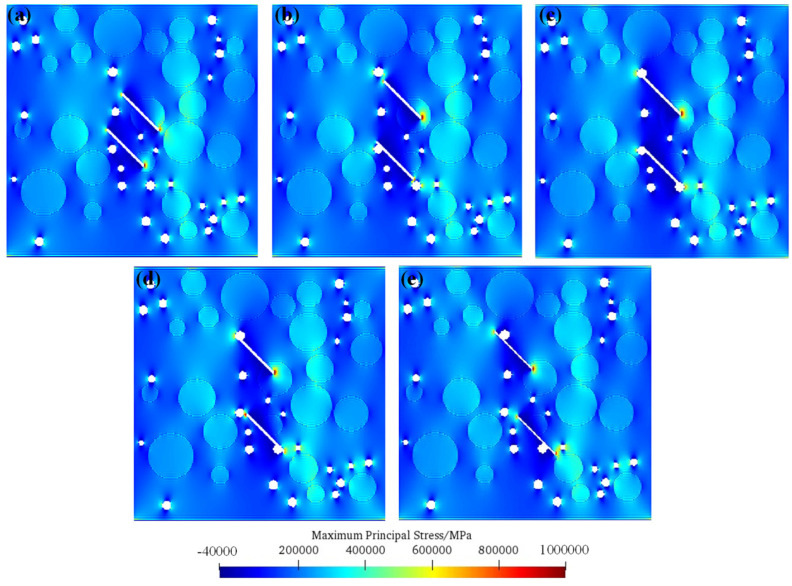
Maximum principal stress distributions before crack initiation in double fissures with different bridge angles. (**a**) *β* = 0°; (**b**) *β* = 30°; (**c**) *β* = 45°; (**d**) *β* = 60°; (**e**) *β* = 75°.

Figure 7 shows the maximum principal stress distributions before crack initiation at different bridge angles of pre-existing micro-fissures. It can be seen from the figure that the tensile stress concentration occurs at the pre-existing micro-fissure tip or the pore overlapping with the pre-existing micro-fissure tip. When the bridge angle of the prefabricated double fissure is small, the tensile stress concentration of the pre-existing micro-fissure tip or the pore overlapping with the pre-existing micro-fissure tip is small, except when the pre-existing micro-fissure tip is inserted into the aggregate. With the increase in the bridge angle, the tensile stress concentration degree of the pre-existing micro-fissure tip or the pore overlapping with the pre-existing micro-fissure tip gradually increases, so the crack is easier to propagate. Therefore, the strength of the double-fissure concrete model gradually decreases with the increase in the bridge angles.

### 5.3. Application Prospects of SPH Method in Concrete Damage Evolution Simulation

In this paper, we propose a simulation method based on the SPH framework for the tensile failure process in fissured concrete, which can effectively reflect the influences of concrete meso-structures (such as pores, aggregates, and transition layers). Compared with the finite element method, SPH is a completely particle-based method, which avoids the need for real-time mesh redivisions during the simulations of crack propagation in the traditional mesh-based method. At the same time, compared with the discrete element method (DEM), the physical parameters in SPH are strictly derived from the partial differential equations, which allows clear physical meanings and avoids the complicated parameter calibration processes of mesoscopic parameters like DEM simulations. Therefore, the SPH method can be effectively applied to cracking simulations for fractured concrete.

However, in this paper, we only reported a numerical simulation for the two-dimensional situation, and the actual concrete structure is a complex three-dimensional shape. Furthermore, the actual aggregate shapes are complex. Therefore, the questions of how to generate polygonal aggregates in SPH, how to reconstruct the complex three-dimensional shapes of concrete by combining computed tomography (CT) scanning technology, and how to introduce it into SPH for three-dimensional calculation while improving the computational efficiency of three-dimensional SPH, comprise the future direction of the SPH method for the simulation of concrete fracture mechanics.

## 6. Conclusions

In this paper, the traditional SPH method was improved to simulate the processes of progressive particle failure. The generation of concrete meso-structures was embedded in SPH. Concrete models containing fissures were established and numerical simulations were carried out. The following conclusions can be obtained:

(1) A simulation method that can reflect the particle fracture processes in SPH is proposed, and the generation of the aggregate, pore, and transition layer in concrete is realized under the SPH framework, which can simulate the meso-tensile failure processes of concrete.
(2) The transition layer, pore, and pre-existing micro-fissures are the weak parts of concrete, which are easy to crack. With the increase in the inclination angle of the pre-existing micro-fissure, the probability of crack growth in the pre-existing micro-fissure decreases gradually, while the probability of crack growth in the pore increases.
(3) Except for the part in the double pre-existing micro-fissure tip, the rest of the crack growth occurs first. Finally, the crack growth generated by the pore and the crack growth generated by the pre-existing micro-fissure tip lead to the final failure of the model. With the increase in the bridge angle, the expansion degree of the crack also increases.
(4) The stress–strain curve for the concrete model presents four typical stages: linear elastic stage, crack propagation stage, failure stage, and post-peak stage. The strength of the concrete model increases with the increase in the inclination angle of the pre-existing micro-fissure, but decreases with the increase in the bridge angle.
(5) The initiation mechanisms of fissured concrete under different schemes are discussed. With the increase in the inclination angle of the pre-existing micro-fissure, the stress concentration degree of the pre-existing micro-fissure that is not embedded in the aggregate end gradually decreases, and with the increase in the bridge angle, the stress concentration degree of the double pre-existing micro-fissure that is not embedded in the aggregate end gradually increases.

## Data Availability

The original contributions presented in the study are included in the article, further inquiries can be directed to the corresponding authors.
